# Development of a 3D Printer for the Manufacture of Functional Food Protein Gels

**DOI:** 10.3390/foods11030458

**Published:** 2022-02-03

**Authors:** Stéphane Portanguen, Pascal Tournayre, Paul Gibert, Selma Leonardi, Thierry Astruc, Pierre-Sylvain Mirade

**Affiliations:** Université Clermont Auvergne, INRAE, UR370 Qualité des Produits Animaux (QuaPA), 63122 Saint-Genès-Champanelle, France; pascal.tournayre@inrae.fr (P.T.); paul.gibert@etu.utc.fr (P.G.); selma.leonardi@etu.utc.fr (S.L.); thierry.astruc@inrae.fr (T.A.); pierre-sylvain.mirade@inrae.fr (P.-S.M.)

**Keywords:** additive manufacturing, personalization, 3D food printing, protein, customization, food design

## Abstract

The use of additive manufacturing is growing in multiple sectors, including food, and its scientific and technological challenges form the subject of much ongoing research. One current hurdle is the implementation of the 3D printing process for meat protein matrices. This article gives an overview of the various 3D printers used to study the printability properties of foods and presents the development of a 3D printer designed to print food protein gels. Printhead development (flow rate and temperature control) and the modifications made to the printing plate (temperature control) are described and discussed in relation to the constraints highlighted in a first prototype. A second, developed prototype was characterized and validated. This last phase showed perfect control of the prototype in the purging of the extrusion system, the flow rate, the calibration and the displacement of the printhead, along with the temperatures at both printhead and plate. A study of the printed gels also revealed good repeatability of the printed gel geometry and pointed to new ways to improve the process. In the near future, the protein gels that will be printed from this prototype will serve as a base for texturizer-free functional foods for people with chewing difficulties.

## 1. Introduction

Additive manufacturing (or 3D printing) is a technology of major interest in multiple sectors including mechanical engineering, medicine, and the food industry [1,2]. For this last sector, two factors can explain the trend: (i) a search for novelty in sensory properties, and (ii) personalization of diet and the design of functional foods for target populations [3], such as older people with chewing difficulties [4,5]. Some authors (e.g., [6]) even affirm that the personalization of diet could result in the printing of food, the formulation and design of which would have been previously adjusted on the basis of data obtained on the specific health status of each individual. To pursue novelty, turnkey commercial printers are available. Several dozen 3D food printers are currently marketed (ChefJet from 3D Systems, Foodini from Natural Machines, Chef3D from BeeHex, etc.). Costs range from a few hundred to several thousand euros/dollars depending on the technical level. Their use is restricted, often being oriented towards culinary design. Nevertheless, these machines are used in many laboratory studies, for example, to determine the rheological or flow properties of food matrices (chocolate, pasta, etc.) or how well the geometry of a printed food corresponds to the original geometry preset in a numerical model [7,8,9]. The range of food printers has been extended to include multi-ingredient machines, which can be used to print more elaborate foodstuffs. However, this type of machine does not ensure a suitable food balance for users because some foods are not printable, such as meat products or most fruits and vegetables. Only products with a low melting point, rich in polysaccharides or naturally gelling can be readily printed (chocolate, biscuit dough, dairy products, cereals, etc.).

To personalize diet and design functional foods of nutritional interest based on proteins of meat origin or/and plant extracts, the right machines have not yet been fully developed. Even so, some studies [10,11,12,13] have focused on the development or modification of prototypes to print a specific single- or multi-ingredient product, but here too, most often of vegetable origin (sugar, chocolate, pasta, etc.). One of the main difficulties lies in controlling the quantity of matrix deposited as a function of the speed of movement of the printhead. However, conventional 3D printers have the advantage of being easily modified and adaptable to the constraints of the food matrices to be printed. The fact that most of them are often open-source and inexpensive is why most laboratory work on 3D food printing is done on this type of machine.

Table 1 summarizes the main printing methods described in the literature together with the main printing settings and variables. These printers are most often designed on the following model: a pressure system is made up of a mechanical (worm screw) or pneumatic syringe pump, with or without temperature regulation, that delivers a set quantity of a semi-viscous food ink through a nozzle or a needle, while controlling the speed of movement and the extrusion rate. A description of the main 3D food printing methods is available in a previously published article [2]. Changes made by users are therefore mainly at the level of the printhead, where the extrusion system is replaced by a type of syringe pump [14]. The printing plates are only very seldom modified. Technically, both commercial food printers and lab-scale modified printers all operate on the same principle of thermal regulation, namely heating of the printhead (as most machines use extrusion) and heating of the printing plate to be able to cook the food if necessary. To our knowledge, to increase the solidification speed of the printed matrices, only forced air cooling systems exist, directly at the nozzle [10]. None of these systems allow the printing plate to be cooled to facilitate mass setting of ingredients. This problem was highlighted by In et al. [15], who found that a mixture of gelatin, sugar, and citric acid could not be printed at 24 °C because the gel failed to set. The authors had to use a device cooled to −2 °C for 1 min between each 0.2 mm layer to increase the firmness of the gel and allow the next layer to be applied. Poor ergonomics coupled with cooling times and general process implementation of this order are incompatible with food-grade 3D printing. This justifies the integration of a self-regulating cooling device directly at the level of the printing plate. Such modified printers are well-suited to research and optimization, but not to the manufacture of food for human consumption because of cleaning and disinfection problems [5]. It is noteworthy that, to date, very few studies have evaluated these health aspects.

Meat products are ill-suited to food 3D printing. The absence of polysaccharides in their matrix impedes mass setting, and the gelling capacity of muscle proteins is limited in the physical and chemical conditions of the raw material (ionic strength, temperature, etc.) [21]. All the currently published studies on animal products, without exception, have used texturizing additives to ensure matrix printability. As an example, Wang et al. [20] printed surimi (containing about 10% starch—average proportion observed, not indicated in the study in question), provided 1.5% sodium chloride was added to solubilize the proteins (even 2.5% for [22]). Yang et al. [23] found the same conclusion with 3D printed chicken meat. According to these authors, a salt addition of 2.5% was necessary for a correct extrusion because it improved cohesion between the printed layers by increasing the water holding capacity and the solubilization of the myofibrillar proteins, thus allowing the structuring of a denser and tighter gel network. Another example is found in the study of Dick et al. [24], who sought to add value to low commercial value muscles by 3D printing. It turns out that printing meat products requires the use of additives such as transglutaminase or guar gum [25] to achieve a viscosity compatible with the printing process. One way to overcome this ultra-formulation is to control the printing process. This is the subject of the present study. The authors describe here how they modified a commercial FDM printer to perfectly control the movement of the printhead and the flow of edible ink at the nozzle, together with the temperatures during printing by thermal regulation at the printhead, but most of all, cold regulation at the printing plate. This last constitutes an original addition to the literature. The authors also studied the effect of these variables on the mechanical strength of a gelatin-based model medium only composed of denatured collagen using this same lab-scale modified 3D printer. Only the feasibility of the machine was investigated in the present study in order to test, in particular, its ability to print a model protein gel in a reproducible way in terms of geometry and texture.

## 2. Materials and Methods

### 2.1. 3D Printers and Extruders

The work presented here concerns the development of a 3D food printer. For this purpose, a first prototype was designed that went on to serve as a basis for the development of a second one, fully described here. The printer supporting the first prototype was a Prusa i3 (Prusa, Prague, Czech Republic). The chosen printer to make into our second prototype was a Ghost1 (Jinhua Flyingbear Intelligent Technology, Jinhua, China). It cost € 320 and was delivered disassembled. This enables the user to discover how the machine works, facilitating future modifications. In this model, the printhead moves along the x- and y-axes. The printing plate ensures the z displacement, leaving ample space to accommodate a custom-designed printhead.

To control the shrinkage of the sample during extrusion (control of the quantity applied), the original extruder (Ghost1) was replaced by a precision volumetric dosing system: Precifluid^®^ (Poly Dispensing Systems, Orgeval, France). With a volume of 10 cm^3^, it has the advantage of being able to produce a liquid or semi-solid material continuously or sequentially, providing a thrust capacity of >500,000 mPa.s, a volume per step of 0.000473 cm^3^, and a minimum delivered volume of 0.06 µL.

### 2.2. Design Method

The extrusion and deposition systems were designed using two approaches. The first approach was the design and production of the parts necessary for the integration of the various essential organs by 3D printing of polymers. All the spare parts used to modify the Ghost1 printer were drawn on the Inventor^®®^ software (Autodesk^®®^, San Francisco, CA, USA). They were then printed with a resolution of 100 μm (nozzle 0.4 mm) in PLA (polylactic acid) or PETG (polyethylene terephthalate glycol) on a 3D Stream30 Pro MK2 printer (Volumic, Nice, France). The slicing software used to generate the G-codes was Simplify3D^®®^ 4.1 (Cincinnati, OH, USA). All the software dedicated to device design was installed on a PC (DELL Precision 3630, Intel Core i9-9900K, 64GB RAM, SSD 1 TB, graphics card NVIDIA Quadro RTX4000).

The second approach was the integration and control of the various components of the 3D printing system described in Section 3.2.2, namely the Precifluid^®^ device, the heating ring, the cooling printing plate composed of the Peltier module and the heat sink, and the optical barrier.

### 2.3. Characterization of the 3D Printer

Major modifications were made to the 3D printer, at both mechanical and electronic levels. It was therefore necessary to make sure that the main components of the device were not negatively impacted by these modifications (e.g., drift of the deposited volume according to the heating temperature of the printhead) and that these modifications yielded gels that were repeatable in shape and hardness.

#### 2.3.1. Effect of Thermal Control of the Printhead on the Flow Rate of the Extruder

The mass flow rate is ensured by the servocontrol of the Precifluid^®^ volumetric dosing system. However, this device undergoes temperature variations of up to 60 °C due to the heat conduction induced by the heating ring used to keep the protein gels supercooled. To ensure the accuracy of the mass flow rate delivered during printing, control of the delivered flow rate was implemented.

As the density of the protein gels was a priori unknown, the tests were performed with water. According to the manufacturer, the Precifluid^®^ system is insensitive to viscosity variations due to its electromechanical movement. One milliliter of water was extruded in 0.2 mL steps at the following temperatures: 20, 40, and 60 °C. Each series of extrusion was repeated 10 times. After extrusion, the quantities of water delivered were weighed using a precision balance (AB204-S, Mettler-Toledo, Columbus, OH, USA) that had been checked and regularly calibrated.

#### 2.3.2. Effect of the 3D Printing Process on the Print Repeatability of Protein Gels

Test gel composition. The gelatin used (type A—200 bloom—13.78% moisture) came from pigskin (Caldic Ingredients, Rognac, France). The water content of the gel was set at 3 kg water/kg dry matter (i.e., for a volume of about 20 mL, 5 g of powdered gelatin plus 16.56 g of ultrapure water). The whole was left at 50 °C, with stirring, until complete dissolution. The gel was poured into the syringe of the Precifluid^®^ dosing system and maintained at 50 °C by the heating ring until final extrusion. Gels were 3D-printed as 20 mm × 10 mm cylinders from an STL format file converted to G-code via ‘Repetier-Host’ software containing the CuraEngine slicer (Hot-World Gmbh & Co. KG, Willich, Germany).

Textural measurements. As the texture of the printed product is a crucial property, it was necessary to ensure the repeatability of the printer in terms of homogeneity of the deposited layers and internal filling. For this purpose, mechanical measurements were made using a texturometer (EZ-Test LX, Shimadzu, Noisiel, France). According to Schreuders et al. [26], instrumental techniques provide objective information on different structural variables and so can be used meaningfully to characterize the structure of meat products. The method chosen for this study was texture profile analysis (TPA) with the following conditions: sample diameter 20 mm, probe diameter 50 mm, surface detection at 0.5 N, displacement speed 20 mm/min, and double compression at 50% of the sample height. Only the hardness values corresponding to the maximum force measured at each compression were exploited. Prior to the measurements, the gel samples were conditioned at 4 °C for 12 h to ensure optimal solidification and then placed at room temperature 2 h before each trial.

#### 2.3.3. Effect of Thermal Gradient (Printing Plate) on the Printing of Protein Gels

The geometry of the 3D printed protein gels was checked using a Leica MZ6 stereomicroscope (Leica Biosystems, Wetzlar, Germany) equipped with a 5.9 M pixel Nikon DS-Fi3 digital color camera. Images and length measurements were made with the NIS-Elements software (Nikon, Tokyo, Japan).

## 3. Results and Discussion

### 3.1. First Prototype—Drawbacks and Limitations

**Figure 1:**
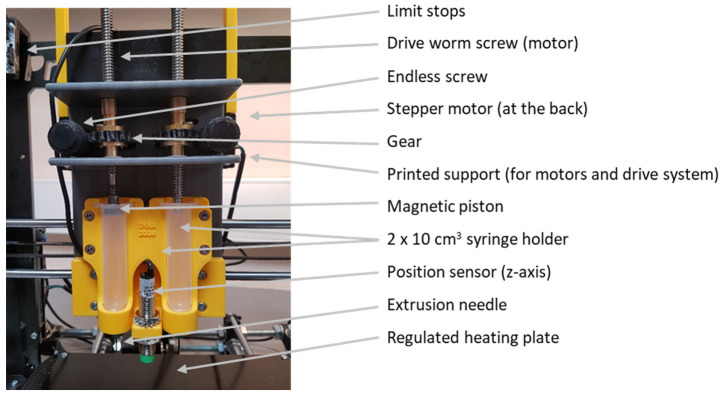
Printhead developed based on a Prusa i3 3D printer. The different modified elements were designed with Inventor^®®^ and then 3D-printed.

The development of the food 3D printer described here was carried out in a second phase. A first prototype was developed [27,28] based on a Prusa i3 3D printer. The printhead of this device, whose major characteristics are presented in Figure 1, enabled us to highlight the main obstacles to the printing of food gels (Table 2). Most commercial 3D printer printheads use stepper motors to extrude the filament. This technique can be adapted and a syringe pump made by replacing the filament drive system with a reservoir equipped with a piston or a worm. In previous work [27], the authors developed a printhead based on this syringe-pusher method, but its limitations soon became evident. Controlling the descent of the piston is delicate because it depends on the matrix used. For relatively viscous gels (gelatin 1.6 g, alginate 1.4 g, phosphate buffer saline 20 mL), the gel holding temperature and shrinkage are problematic. Despite reversal of the motor rotation to ensure the displacement of the piston, a residual flow of the matrix occurs for several minutes owing to the pressure built up inside the syringe. This makes it difficult, if not impossible, to control the geometry of the printed object.

### 3.2. Second Prototype

The Ghost 3D printer was modified to allow printing of protein gels by adapting the process to the matrix. Modifications were made to the control of the prototype, to its head, and to its printing plate.

#### 3.2.1. Hardware Control

**Figure 2:**
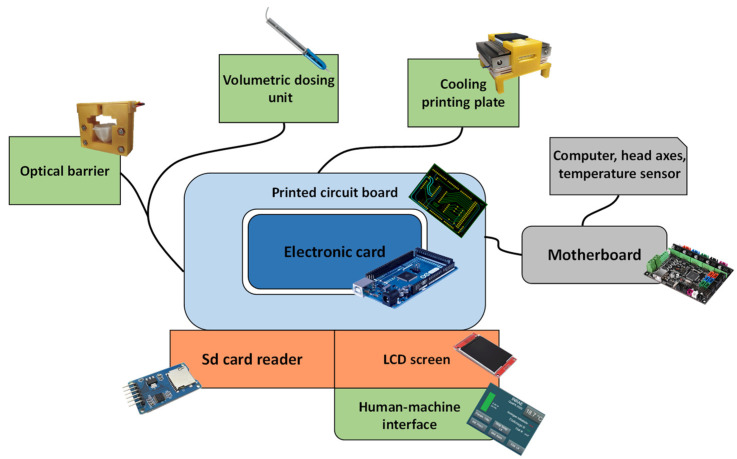
Schematic representation of the various electronically controlled components of the 3D food printer built. “Supplementary Materials S1” gives accurate information about the connectivity between the different added elements.

New hardware elements were integrated (Figure 2). These included a volumetric dosing system (for accuracy of deposition), a thermal regulation of this dosing system (to keep the matrix supercooled), a Peltier cooling module (to assist in gel setting), a series of sensors (control/driving), and an optical barrier (extruder purge). To avoid adding constraints or major modifications to the existing motherboard, a new electronic control board was also added. This board allows the new elements to be interfaced with the motherboard. Communication between the two boards is essential to keep the actions of each board synchronized.

The new electronic board (Arduino Mega 2560 type microcontroller board programmed in C) was accompanied by a printed circuit board whose role was to interface the board with the rest of the elements and to house the electronic components, a touch-sensitive LCD screen for managing the “human-machine” interface, and an SD card reader for storing the printer information and operating variables. The volumetric dosing system and the cooling plate were the two main elements added. A light barrier and a “human-machine” interface created specifically for the use of this printer facilitated its use.

The communication between the original motherboard and the added electronic board was at first done in a binary way using available digital inputs and outputs. However, this binary mode of communication between the two boards soon proved insufficient. It failed to allow the transmission of more complex information for the operation of the printer such as the temperature measured by the motherboard or the position of the various axes of the printer. The use of a screen managed by the electronic board allows the use of a UART (universal asynchronous receiver-transmitter) serial link. This type of communication requires two data wires: one for sending bytes (TX) and another for receiving (RX). It is thus possible to send and receive data at the same time due to the bidirectional asynchronous link. A third wire is used to set the same electrical potential reference for both cards, which is mandatory to read the data sent or received. Communication by UART link is usually done by sending information (sentences, numbers, etc.) coded in ASCII (American Standard Code for Information Interchange) decimal format. In the case of the printer motherboard, communication with external devices took the form of G-code modified by the Marlin firmware. The G-code was supplemented by M-code, which allowed control of the machine and its specific features. By connecting the motherboard and the electronic board via the UART-type serial link, it was possible to enable communication by exchanging G-code. However, the electronic board must be programmed to understand the ASCII coding and to interpret the G-code instructions used by the motherboard firmware. 

Unlike the Arduino Mega 2560 board, the motherboard has only one UART-type serial link, linked to two different physical outputs. One is connected to the USB port, used for communication with the computer, and the other to the wired connections (RX and TX) linked to the Arduino electronic board. As the motherboard is unable to identify the source of the information received, using both outputs at the same time can lead to communication errors. To limit errors, it is essential that the motherboard, the electronic board, and the computer’s USB port exchange and process information at the same speed. This speed must be set by the maximum value usable by the Arduino board, which is lower than that of other equipment; in practice, the “baud rate” is limited to 115,000 bits per second. In addition, the Repetier-Host software is unable to initiate communication with the motherboard when the transmitter wire (TX) of the Arduino board is connected to the serial link, even if this wire is inactive. As it is impossible to deactivate this wire by computer without breaking the connection between the Arduino board and the motherboard, it was necessary to set up a static relay to cut the connection between the transmission wire and the motherboard. This static relay was controlled by a digital output of the Arduino board, and only allowed connection between the transmission wire and the motherboard when information had to be sent to the motherboard.

The flow rate is a fundamental variable in obtaining a correct extrusion. In our working configuration, this flow rate was determined by both the slicer of the software and the operator. In the slicer, the flow rate was calculated according to the geometry, the line width, and the nozzle size. The flowrate was thus directly correlated with the movement of the printhead. Faster movement will extrude faster to ensure the deposition of the right amount on each circle arc. Normally, no flow adjustment is required, in particular when printing polymers to which the software used is well-suited. However, as our gels behaved differently from polymers, manual flowrate adjustments were sometimes needed to ensure cohesiveness depending on the wall thickness, layer height, infill rate, etc. This was handled by manually adjusting the extrusion multiplier setting available in the CuraEngine slicer of the Repetier-Host software.

#### 3.2.2. Printhead Design

**Figure 3:**
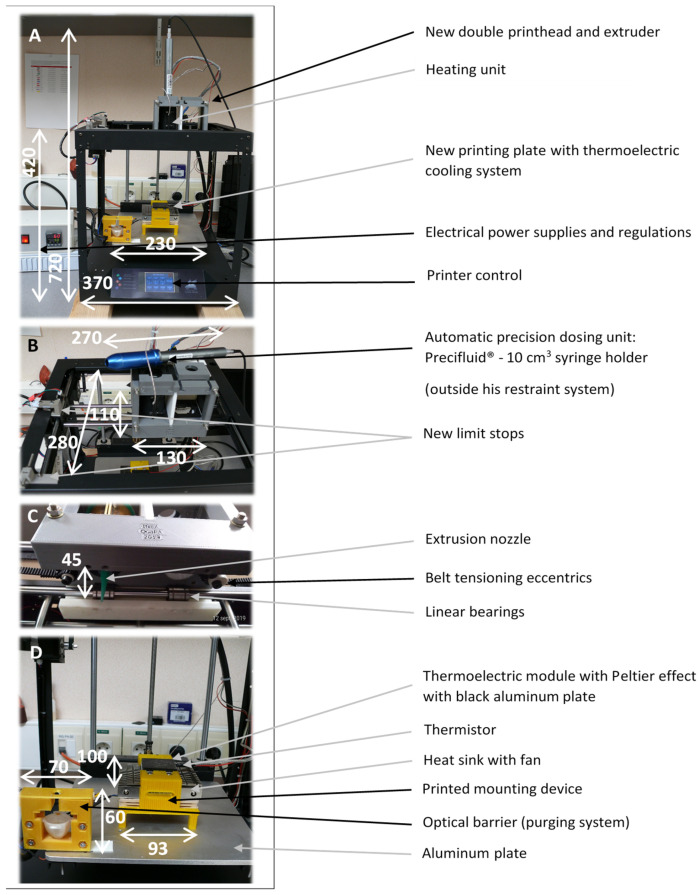
(**A**) General view of the lab-scale modified Ghost printer. (**B**) Top view of the printhead with the volumetric dosing system removed. (**C**) Bottom view of the printhead. (**D**) View of the new printing plate (usable area 16 cm^2^). Dimensions are in mm. The drawings of the specifically-designed parts for modifying the 3D printer are available in Supplementary Materials S2.

To overcome the uncontrolled shrinkage problems stated in the section on the first prototype, it was decided to couple the Precifluid^®^ system to the printer (Figure 3A,B). This system has the advantage, thanks to the extremely precise control of its motor, of being able to deposit matrices of very different viscosities, ranging from water up to the gel stated above. However, as this is a commercially available device intended for stand-alone use, its control had to be completely redesigned.

To control the volumetric dosing system, the use of a new driver circuit for the Precifluid^®^’s stepper motor eliminates the need for a control box. To physically integrate the Precifluid^®^ system into the printer, a device was designed and then printed in PLA (Figure 3A–C and “Supplementary Materials S2”). This allowed the Precifluid^®^ system to be mounted and dismounted ergonomically by simply sliding it in and out of the printer for the filling and cleaning phases or even to change consumables. This last phase is very seldom optimized on lab-scale modified 3D printers. As shown in Figure 3B, this device comprised two mobile parts allowing the Precifluid^®^ system to be held in place, and it was possible to install two volumetric dosing devices on the printhead to increase the number of ingredients used to print the food. Sufficient space was also reserved for the installation of a brass heating collar delivering a power of 6.5 W.cm^−2^ (Acim Jouanin, Evreux, France), to enable the temperature of the useful part of the volumetric dosing system (10 cm^3^) to be regulated. To achieve this, an aluminum sleeve was specially machined to fit both on the external face of the Precifluid^®^ system up to the tip of the nozzle (to avoid clogging due to gel solidification), and on the internal face of the heating collar, thus allowing optimal thermal conductivity between the two elements. The temperature regulation of the heating collar was ensured by a calculation based on a PID algorithm and controlled by a K-type thermocouple, previously calibrated using a reference platinum probe (model Testo 735, Testo, Forbach, France) placed on the external face of the aluminum sleeve. The collar was heated at least 30 min before printing to ensure temperature homogeneity.

The original volume of the printhead was increased by a factor of four compared to the original head. The limit switches were therefore relocated and mounted on new 3D printed stainless steel supports. Linear guides were also added for *x*-axis movement (Figure 3C).

Before each printing, an initialization phase of the Precifluid^®^ system was programmed to purge the syringe and eliminate any air bubbles due to the filling of the syringe and to prevent any clogging at the nozzle outlet. For this purpose, the printhead was placed at the *x-* and *y*-axis stop. The Precifluid^®^ system then extruded the ink through a 3D printed device equipped with an optical barrier. When the optical beam was cut by the extruded fluid (700 nm wavelength laser diode), the pusher stopped to keep the gel in a compressed state, preventing the formation of a new air bubble. The syringe was thus ready to be used for printing, and gel extrusion will be immediate. The threshold for fluid detection by the optical barrier was determined experimentally to optimize the removal of air bubbles while minimizing gel loss. 

#### 3.2.3. Printing Plate Design

The original Ghost1 printer plate was replaced by a 5 mm-thick aluminum plate. On this plate was fixed a PETG printed device (Figure 3D) designed to contain a 51.4 W Peltier effect module (Laird Technologies, Chesterfield, MO, USA) resting on a heat sink (NH-L9i cooler pack, Noctua, Vienna, Austria) to avoid overheating of the system. This heat sink was equipped with a 12 V electrically powered fan with a flow rate ranging from 1.13 to 1.61 m^3^.min^−1^ (Figure 3D).

The 16 cm^2^ cross-sectional printing surface was composed of the Peltier effect module on which was positioned the 5 mm-thick aluminum plate of cross section 40 × 40 mm to ensure optimal thermal conduction. A 3.5 mm-deep groove was machined in the center of this plate to seal a 100 kΩ thermistor (Zhuhai Bell Technology, Zhuhai, China) as close as possible to the printing surface. The thermistor was sealed into the groove using a conductive resin loaded with 80% aluminum particles. The thermistor, whose accuracy was ±0.5 °C, was regularly calibrated at five points between 0 and 100 °C using a platinum reference probe (model Testo 735, Testo, Lenzkirch, Germany). The contact between the aluminum plate and the Peltier module was made through the yellow PETG device (Figure 3D) by means of a layer of conductive grease added between the two. The Peltier module was controlled by the Arduino board using a PID algorithm to limit any temperature disturbances induced (e.g., by the deposition of hot gel). The Peltier module’s power was provided by chopping a 12 V output from an external power supply (12 V, 33 A) able to deliver a much higher power than that consumed by the Peltier module. Chopping the 12 V supply with the Arduino board from the result of the PID regulation algorithm made it possible to vary the average supply voltage of the Peltier module between 0 and 12 V, thus enabling the optimization of the cooling to reach the temperature setpoint.

Before each use, the printing plate position was calibrated at nine points. This calibration was done by requesting the Marlin firmware of the motherboard by sending it the G-Code command G29. From this command, the Marlin firmware took full charge of the calibration of the printing plate. This calibration consisted in moving the printhead’s *z*-axis position sensor to nine points evenly distributed over the surface of the plate and measuring the deviation from the *z* = 0 position. Thanks to the automatic Marlin correction, this calibration made it possible to avoid horizontal defects in the plate and thus to guarantee an identical print height over its entire surface.

### 3.3. Characterization of the 3D Printer Components

#### 3.3.1. Printing Plate Temperature Regulation

As the cooling plate was now equipped with a temperature sensor, it was necessary to characterize its behavior in terms of regulation speed, particularly when subjected to a deposit of hot material. The main objective was to ensure that it was efficient enough for the first layers deposited to be cooled very quickly.

**Figure 4:**
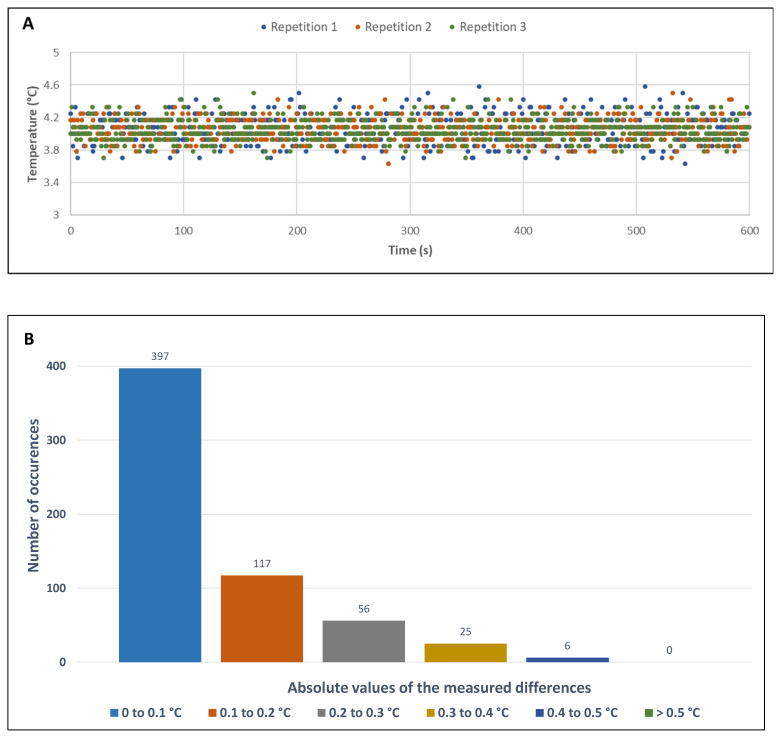
Graphical representation illustrating the temperature control of the printing plate. (**A**) Stability over 600 s for a setpoint temperature of 4 °C and one measurement per second. (**B**) Data distribution according to the absolute value of their deviation from the setpoint.

First, the temperature setpoint was set at 4 °C and the temperature measured by the thermistor was recorded for 600 s, the average time for printing small model foods. Figure 4A shows a very satisfactory regulation over three repetitions of these recordings performed at a frequency of 1 Hz. All samples were distributed over a range of 1 °C, between 3.6 °C and 4.6 °C. The average of each repetition was 4.04 ± 0.17, 4.05 ± 0.14, and 4.04 ± 0.13 °C, respectively. Figure 4B, which ranks the recorded temperature values according to their deviation from the setpoint, shows that two-thirds of them were below ±0.1 °C. Identical results were obtained for a setpoint temperature of 10 °C.

**Figure 5:**
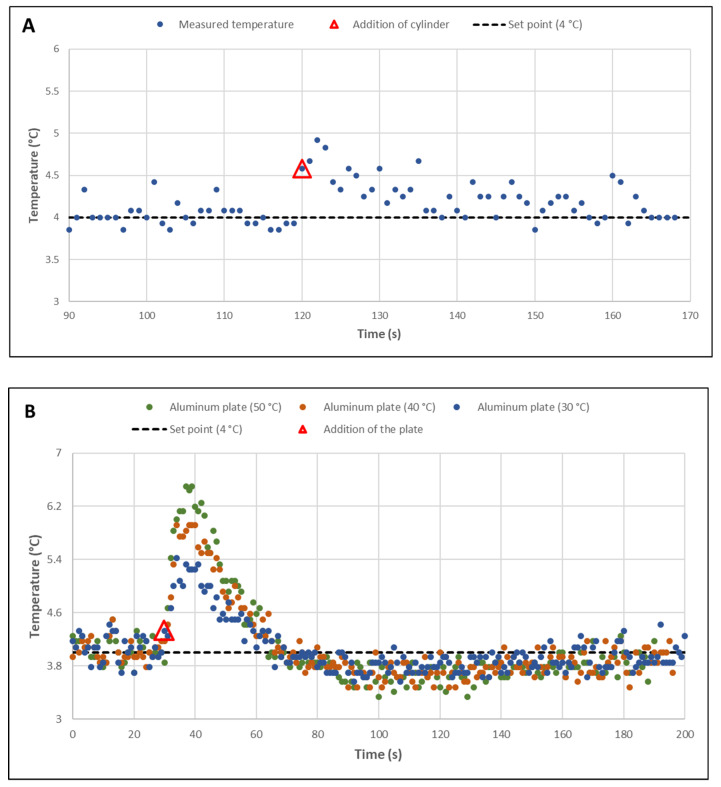
(**A**) Visualization of the temperature deviation from the setpoint after a hot gelatin cylinder (30 °C) 20 mm in diameter and 10 mm high was placed on the 16 cm^2^ printing plate. (**B**) After placing an aluminum plate 2 mm thick and 16 cm^2^ in cross section heated to 30 °C, 40 °C, and 50 °C on the printing plate.

The second characterization step consisted in evaluating the temperature compensation by the PID-type control during the deposition of a hot mass. This could simulate the deposition of material during extrusion. For this purpose, a gelatin cylinder 20 mm in diameter and 10 mm high, containing 3 kg water/kg dry matter and conditioned at 30 °C, was deposited on the printing plate after it had been stabilized at 4 °C for 120 s. The temperature increase due to the deposit was very small and hardly distinguishable from the temperature variations due to the regulation (Figure 5A). To check the stability of the regulation at higher temperatures so as to approach a food-grade printing temperature, a 2 mm-thick aluminum plate, equal in size to the printing plate, and heated to 30 °C, 40 °C, and 50 °C, was positioned after 30 s (i.e., the time required for the plate temperature to be stable). This aluminum plate was chosen for this experiment because at the temperatures tested, the protein gels are in the liquid state and could therefore spread out over the printing plate. These unfavourable conditions in terms of contact surface and mass were thus chosen to check how the control operated. The results presented in Figure 5B show a very dynamic response of the regulation. A significant increase in the temperature of the plate was recorded 10 s after the deposition, and the target temperature was reached again after 40 s with an observed difference of −0.5 °C, which disappeared after a further 60 s. Under normal operating conditions, such a large mass cannot be deposited on the plate all at once, as the deposition during printing is carried out in successive layers, forming a thickness of 0.2 mm and with a maximum width corresponding to the diameter of the nozzle used.

**Figure 6:**
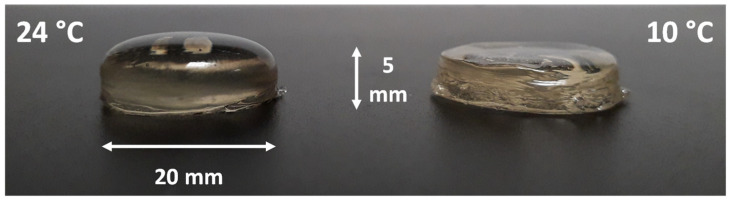
Effect of printing plate temperature on the cylinder geometry of protein gels.

To assess the importance of the cold regulation of the printing plate for the printing of animal protein gels, tests were carried out at 24 °C (room temperature) and 10 °C. The temperature of 10 °C was determined experimentally in preliminary tests and identified as best for printing this type of gel. Below this value, the gels solidified too rapidly and the deposition of layers took place irregularly. Above this value, the solidification was insufficient and the gel flowed without conforming to the programmed geometry. Figure 6 shows the effect of the printing plate temperature on the final shape of the printed object at room temperature. At 10 °C the shape was much more regular, whereas at 24 °C the printed shape was cylindrical only at its base and formed a dome. Note that the printed objects were deliberately 5 mm high to avoid the effect of the thermal gradient explained in Section 3.4.1.

#### 3.3.2. Effect of Temperature on Extrusion Rate

Following the modifications made to the printer and to the Precifluid^®^ system control, it was now possible to adjust the extrusion rate to between 0.001 and 0.610 mL.min^−1^, for a nozzle diameter of 0.69 mm. The use of a larger nozzle diameter allowed an increase in the flow rate. 

**Figure 7:**
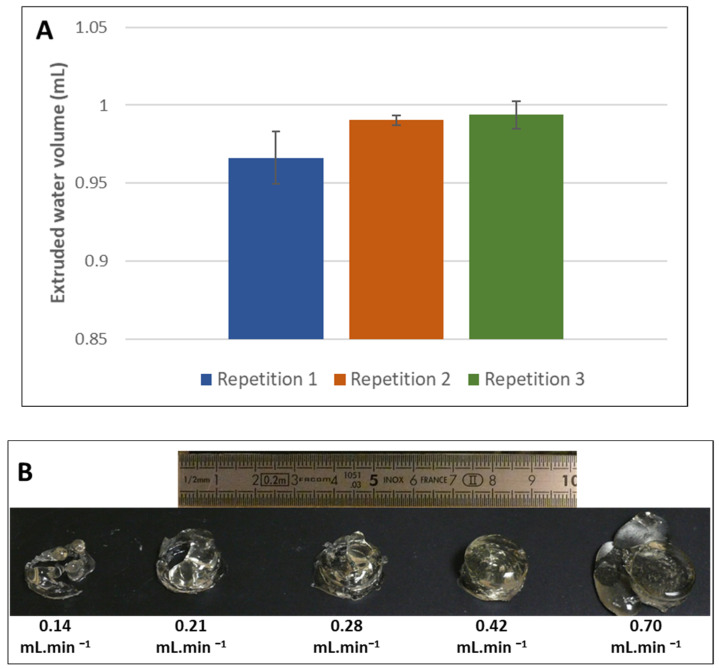
(**A**) Verification of the volume control delivered by the extruder at 60 °C. (**B**) Effect of flow rate on the geometry of the printed object.

Figure 7A shows the results obtained at 60 °C, highlighting, for the three repetitions, measured deviations ranging on average from 0.01 to 0.03 mL (i.e., 1–3%), which was perfectly acceptable given the operating conditions of the extruder. We note that the values for test 1 were lower and presented a higher standard deviation, which can be explained by the presence of an outlier (17% deviation instead of 1–3%) among the 10 repetitions of the test, probably due to an air bubble in the extruder.

Figure 7B shows the effect of the flow rate on how well the predefined geometry of the object to be printed was followed, here, a gelatin gel cylinder 20 mm in diameter and 10 mm in height. This form corresponds to a portion that could serve to supplement a patient with protein deficiencies. A flow rate of 0.42 mL.min^−1^ gave a shape visually very close to the original geometry. Gelatin has a rheofluid behavior (i.e., as flow velocity and shear rate increase, its viscosity decreases). This behavior can be observed in Figure 7B: for a flow rate of ≤0.42 mL.min^−1^, the viscosity of the gelatin was too high to allow sufficient flow (the flow rate was reduced) and so conformity to the preset geometry was lost. Conversely, for a flow rate of ≥0.70 mL.min^−1^, the viscosity was lower, the flow was greater, and the geometry was again lost. These findings make it possible to relate the process settings to the molecular nature of the printed matrix. Materials such as gelatin have an organized structure based on long-chain molecules that resist flow. As the shear rate increases, the molecular chains line up in parallel and tend to slide over each other. The resistance to flow and the viscosity are then lower [29].

### 3.4. Characterization of the 3D-Printed Matrices—Effect of the Process on the Geometry and Texture of Protein Gels

#### 3.4.1. Geometry

**Figure 8:**
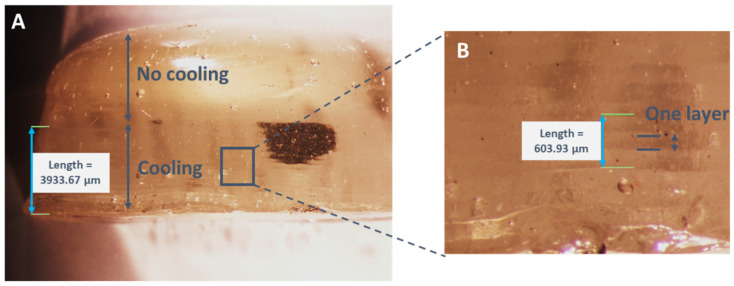
Effect of thermal gradient on the geometry of protein gels. (**A**) Examination of the printing defect due to lack of cooling. (**B**) Control of the thickness of the deposited layers.

The various experiments revealed heterogeneity in the geometry of the printed gels along the *z*-axis. This was initially attributed to a problem in settings during 3D printing, particularly for the extrusion rate. However, observations with a stereomicroscope (Figure 8A) revealed a probable link between the shape of the object and the presence of a temperature gradient within the object itself. Figure 8A shows a regular height of the gel deposits over about 4 mm, with the printing layers visible. From 4 mm onward, a curvature can be observed that deformed the upper part of the object (dome in Figure 6), the initial cylindrical geometry being lost. This can be attributed to the printing plate cooling system, i.e., the temperature of the deposited layers was no longer cold enough beyond 4 mm for layer *n*-1 to set rapidly and so allow a satisfactory deposition of layer *n*. This problem had been envisioned during the design of the device without it being possible to predict the height of the thermal gradient. This finding further justifies a second temperature control system (currently under development) to compensate for the temperature rise within the sample, which acts as a thermal insulator with respect to the printing surface. However, Figure 8B shows that the thickness of the deposited layers was perfectly in line with the value requested in the slicer: 0.2 mm (603.93 µm measured for three layers, i.e., 201.31 µm on average per layer).

Weighing was also done to check the repeatability of the gel printing. For five samples printed under the same conditions, the average mass was 1.944 ± 0.035 g, i.e., a variation of ±1.79% of the total sample mass. In addition to the good repeatability of the geometry of the printed gels, a good repeatability of their mass was also obtained, indicating good repeatability in terms of filling the printed gel. These measurements confirm that the purge programmed before the printing step is effective and necessary to limit the number of potential air bubbles.

#### 3.4.2. Textural Measurements

**Figure 9:**
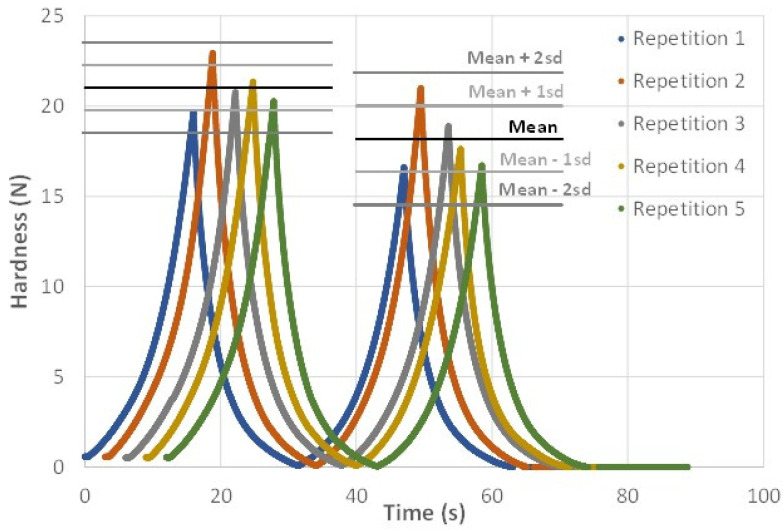
Hardness measurements of printed protein gels using the TPA test. The horizontal line represents the mean (in black) of the maximum forces for the five samples, and for the two successive compressions. The standard deviations around this mean are represented by the shaded horizontal lines.

Five cylinders of protein gels were printed at a flow rate of 0.42 mL.min^−1^. The hardness of each gel was assessed by TPA measurement and compared to that of the other gels. Figure 9 shows the double compression curves obtained for the five gels tested. To facilitate visualization, each curve has been shifted from the previous one by 3 s along the *x*-axis. The mean and standard deviation of the maximum values of each peak were calculated and appear on the graph as horizontal lines, showing a very good repeatability of the measurements. Four out of five repetitions are within ±1 standard deviation (sd) and all repetitions are within ±2 sd.

## 4. Conclusions and Perspectives

This study shows that it is possible to modify a commercial FDM 3D printer to finely control the printing variables of a protein gel and ensure the printing of a homogeneous gel. The impact of these modifications on the printing of the protein gels was evaluated after significantly modifying the main components of the printer and its control. It was found that the modifications to the device resulted in gels of repeatable size, layer thickness, and hardness. The adjustment of the flow rate, and therefore the shear rate, and the control of the plate temperature during the printing phase, which are essential to ensure that the geometry of the printed food is maintained, were fully controlled. Although maintaining the printing plate temperature at 10 °C was effective in guaranteeing the mass setting of the gels and made it possible to control the texture of the matrix without having to use texturizing additives, the presence of a temperature gradient over the first few millimeters of the gel height still requires the development of a second cooling device.

The present study can serve as a starting point for the development of foods adapted for older people and more broadly for people with chewing deficiencies. As the older population, often affected by sarcopenia, is expected to increase considerably in the coming decades, alternatives to current protein foods, which are often difficult to chew, are needed. Future studies will exploit the benefits of controlling the printing variables described in this study, by combining them with a natural biochemical reaction to improve food texture. The synergy of these two approaches should enable marked progress in controlling the texture of foods designed for people with chewing deficiencies.

Work remains to be done on the prototype described here, particularly to address health and safety aspects, both microbiological and chemical. New metal 3D printing technologies offer new design opportunities. The possibility of printing devices made of 316L stainless steel, coupled with surface treatments designed to limit the formation of microbial biofilms, makes it possible to envisage direct contact with food in compliance with regulations. Future developments will therefore need further improved control of the 3D printing process by adding an additional cooling system to counter the temperature gradient within the food, which is created as the food is printed on the plate. The design of the printhead can also be revised using metal 3D printing technologies and generative design, so that all these new functionalities, including post-processing, can be integrated into one device. Generative design is an iterative process that optimizes the geometry of an object, considering a number of constraints specific to the object itself. This process thus makes it possible to limit the quantity of material used and so lighten the object while ensuring a robust structure [30]. In the case of the development of printheads for edible inks, this method can greatly help in the arrangement of the supply systems for the various fluids necessary during the extrusion of the edible ink, during the cooling of the printed food, and even during its post-processing. The parts resulting from the generative design will have to be integrated into the global process without causing disturbances, e.g., in the temperature control. This is why studies concerning the modelling of heat and mass transfers [31] or the digitalization of phenomena with the help of a digital twin can provide real added value [32].

## Figures and Tables

**Table 1:** Main methods of 3D printing of food products and their associated settings and operating variables. Only studies with numerical data have been included.

Printing Technology	Type of Food	Print Speed (mm.s^−1^)	Travel Speed (mm.s^−1^)	Extrusion Rate (mL.min^−1^)	Nozzle/Needle Diameter (mm)	Infill Level (%)	Layer Thickness (mm)	Ref.
Syringe pump made of stainless steel (1.3 b pressure)	Mixture of fruits/vegetables + fish collagen	11–21	20	/	1.2	25	1.1	[5]
Syringe pump	Sugar	20–50	/	/	1	/	/	[10]
Coaxial extrusion	Pectin/CaCl_2_	10	200	0.34	0.838	85	0.838	[14]
Syringe pump	Mashed potatoes/strawberry juice gel	25	/	/	/	40–100	1.2	[16]
Syringe pump	Various foods (viscosity 1.1.10^−3^ to 10^3^ Pa.s)	15–20	15–20	/	/	/	/	[17]
Piston (4 b pressure)	Cereal dough	30	50	/	0.6	10–20	0.3–0.5	[18]
Auger mixer + conveyor	Lemon juice gel + potato starch	30	/	1.44	1	/	/	[19]
Auger mixer + conveyor	Surimi	28	/	0.18	2	/	/	[20]

**Table 2:** Main advantages and disadvantages of the first printhead prototype developed from the Prusa i3 3D printer. ^1^ Original parts of the 3D printer and, ^2^ Modified devices for the present study.

Original Parts ^1^/Modified Devices ^2^	Advantages	Disadvantages
Printhead ^2^	Double extrusion	Displacement limited to 20 mm in *z*-axis
Extruder ^2^	Simple design	Poor retraction
Syringe holder ^2^	Adapted to the printing volume	Non-ergonomic
Printing plate ^1^	No modifications	No cold control

## Supplementary Materials

The following supporting information can be downloaded at https://www.mdpi.com/article/10.3390/foods11030458/s1, Supplementary Material S1A: Graphic of the Hardware Connection; Supplementary Material S1B: Graphic of the Process; Supplementary Material S2: Elements added to the original 3D printer, designed by Computer aided design (Autodesk Inventor 2019) and manufactured by 3D printing (excluding part B2 which is machined from aluminum 2017A). Dimensions are in mm.
