# Supplementary material for: Development of a 3D Printer for the Manufacture of Functional Food Protein Gels
Source: Foods. 2022 Feb 3;11(3):458. doi: 10.3390/foods11030458 (PMC8834083; doi:10.3390/foods11030458)
Supplement: Supplementary file 1 [file foods-11-00458-s001.zip › foods-1530161-supplementary.pdf]

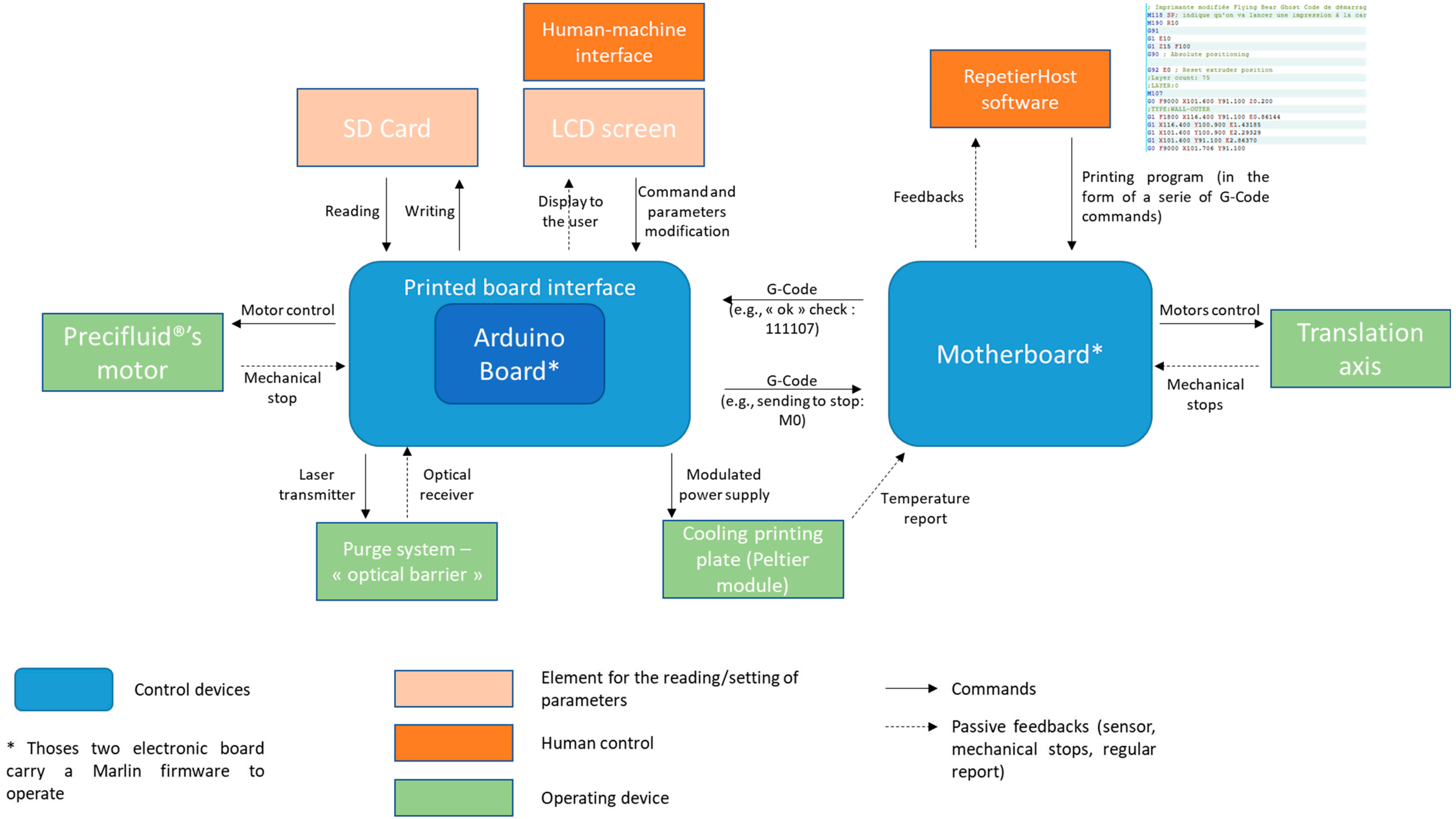
Human-machine interface
SD Card
LCD screen
RepetierHost software
; Imprimante modifiée Flying Bear Ghost Code de démarrag
M118 SP; indique qu'on va lancer une impression à la car
M190 R10
G91
G1 E10
G1 Z15 F100
G90 ; Absolute positioning
G92 E0 ; Reset extruder position
;Layer count: 75
;LAYER:0
M107
G0 F9000 X101.600 Y91.100 Z0.200
;TYPE:WALL-OUTER
G1 F1800 X116.400 Y91.100 E0.86144
G1 X116.400 Y100.900 E1.43185
G1 X101.600 Y100.900 E2.29329
G1 X101.600 Y91.100 E2.86370
G0 F9000 X101.706 Y91.100
Reading
Writing
Display to the user
Command and parameters modification
Feedbacks
Printing program (in the form of a serie of G-Code commands)
Printed board interface
Arduino Board*
G-Code (e.g., « ok » check : 111107)
G-Code (e.g., sending to stop: M0)
Motherboard*
Motor control
Precifluid®'s motor
Mechanical stop
Motors control
Translation axis
Mechanical stops
Laser transmitter
Optical receiver
Modulated power supply
Temperature report
Purge system – « optical barrier »
Cooling printing plate (Peltier module)
Control devices
* Thoses two electronic board carry a Marlin firmware to operate
Element for the reading/setting of parameters
Human control
Operating device
Commands
Passive feedbacks (sensor, mechanical stops, regular report)

<u>Supplementary Material S1A:</u> Graphic of the Hardware Connection

The whole printer is driven by two control devices: the motherboard (from the original printer) and an additional Arduino board. The communication between those two control devices is done through a UART serial link. The two boards send instructions (e.g., the stop command « M0 » or launch a program) and information (e.g., temperature report or modification of a parameter value) to each other's serial ports in ASCII language. We programmed our Arduino to understand the ASCII message that it receives and to send instructions to the motherboard in G-Code.

The motherboard commands the features that were in the original printer and that were unchanged (motors for the XYZ translation, bed temperature report). Regarding the Arduino, it controls the operating devices that we added to the printer or that we modified (cooling Peltier module, extruder, optical barrier). For both cases, the electronic boards send commands to motors or power devices and receive feedback (mechanical or optical stops). In the case of the cooling printing plate, the temperature feedback is sent to the motherboard by the thermocouple of the original printer. The motherboard then sends this temperature report to the Arduino board. The power supply of the cooling Peltier module is modulated through a static relay that is controlled by a PWM signal (pulse width modulation). This PWM is itself modulated according a PID algorithm of the error to the targeted temperature.

Reading and setting of parameters are handled by different elements. A SD card is used as a back-up to keep some parameters from one print to another (screw position of the extruder, nozzle diameter, etc.). When the printer is turned on, the first step is to read parameters from the SD card. At the end of a process (a program or a manual action), new values of the parameters are written on the SD card.

The human-machine interface is borne by the LCD Screen and the Repetier software. The LCD screen is used to manually change some parameters (nozzle diameter, axial speed for manual translation) or to send commands to the Arduino board (manual extrusion, launching of initialization program). On the other hand, the Repetier software generates a printing program (in the form a series of G-Code instruction) that sets the printing parameters and the commands to the operating devices. Both of them display feedback to the user.

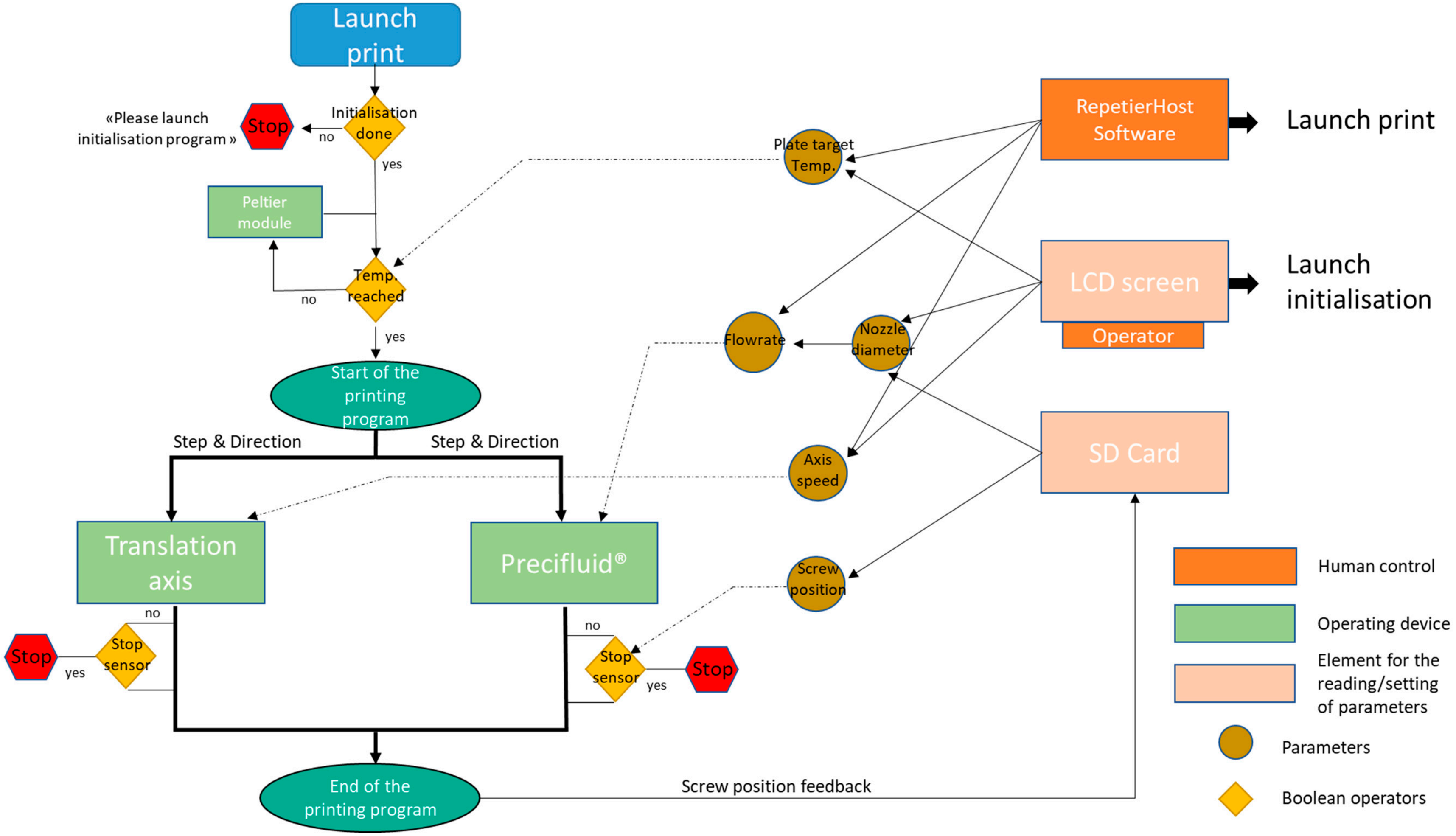
Launch print
Initialisation done
no
yes
Stop
«Please launch initialisation program»
Peltier module
Temp. reached
no
yes
Start of the printing program
Step & Direction
Step & Direction
Translation axis
Precifluid®
no
no
Stop sensor
Stop sensor
yes
yes
Stop
Stop
End of the printing program
Screw position feedback
Plate target Temp.
Flowrate
Nozzle diameter
Axis speed
Screw position
RepetierHost Software
Launch print
LCD screen
Operator
Launch initialisation
SD Card
Human control
Operating device
Element for the reading/setting of parameters
Parameters
Boolean operators

<u>Supplementary Material S1B:</u> Graphic of the Process

The programs are launched by human action: the initialization program is launched from the LCD screen and the printing program, from Repetier software. Pressing a button on the screen or on Repetier send a G-Code command to the motherboard and the Arduino board so that they start the desired program.

Printing program consists beforehand of a series of Boolean operators (*yellow diamonds on the graphic)* to check the status of different parameters (e.g., that the bed has reached the desired temperature or that the initialization procedures have been realized before starting a print). A series of G-Code instructions is then sent to the motors of translation and extrusion. At any point of the process, if a mechanical stop is pressed, a stop command is sent to both the Arduino board and the motherboard and prematurely ends the program.

The initialization program is not represented here but it also works with Boolean checks, then s series of instructions to the motors to conduct the purge program and the tray calibration program. The purge system is designed to get the screw of the Precifluid® in contact with the piston of the syringe and get the syringe ready to extrude without losing too much gel. The syringe extruder goes above the optical barrier and starts activating the motor. The optical barrier consists of a laser transmitter pointed toward an optical receiver. When the gel is finally being extruded, it cuts the laser beam. The lowering of the signal of the optical receiver ends the initialization program. Regarding the tray calibration, we use the program already implanted in the motherboard (G-code command: G29).

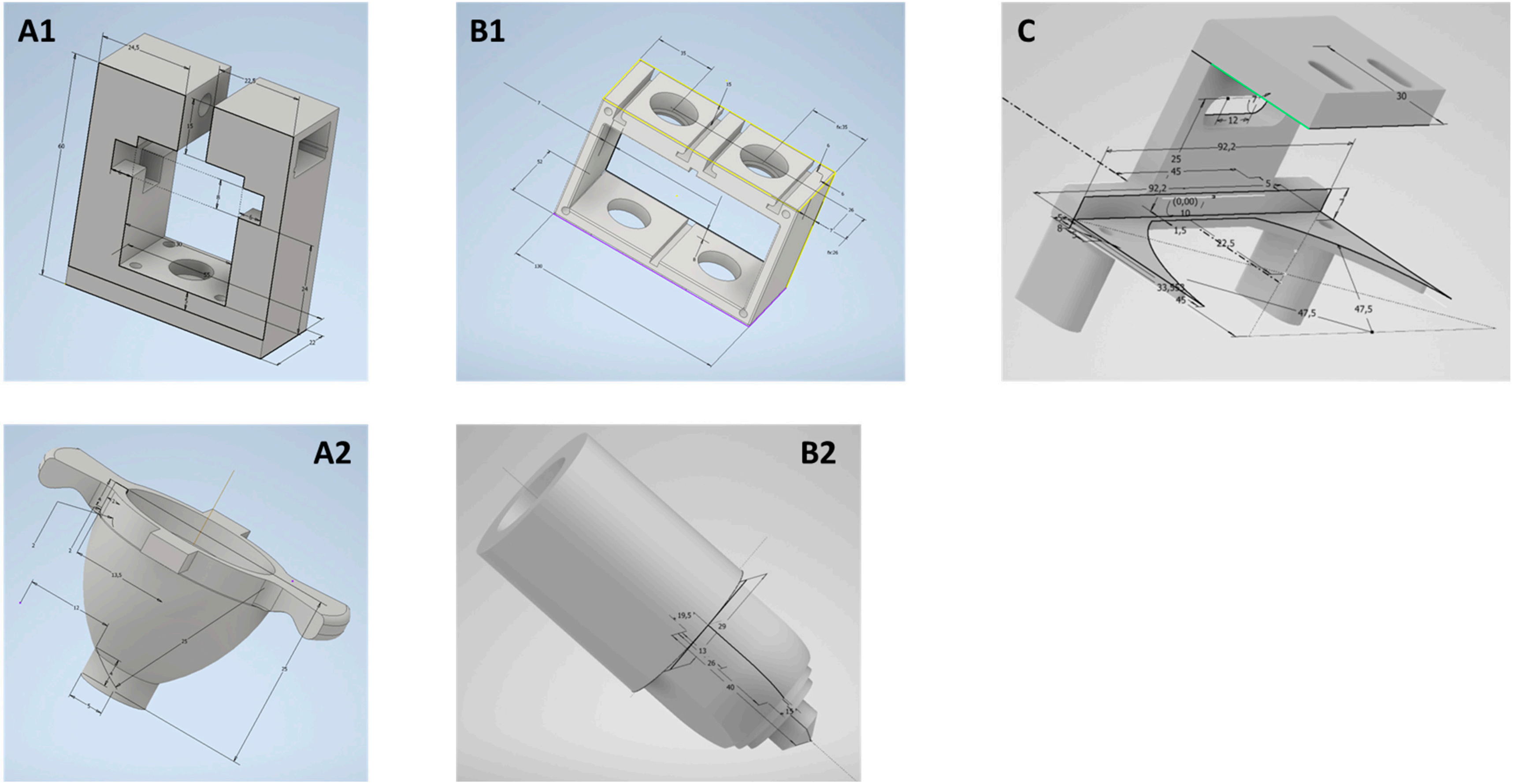

<u>Supplementary Material S2:</u> Elements added to the original 3D printer, designed by Computer aided design (Autodesk Inventor 2019) and manufactured by 3D printing (excluding part B2 which is machined from aluminum 2017A). Dimensions are in mm.

A1: Light barrier support in which the laser diode and the receiver are placed; A2: Removable receptacle for purged gel; B1: Extrusion system support; B2: Aluminum sleeve in which the Precifluid® is placed for an optimal thermal contact; C: Support for the cooling plate and the heat sink. Only half of the support is showed.
